
JOURNAL INFORMATION
==============================
NLM Title Abbreviation: ScientificWorldJournal
Journal ID: ScientificWorldJournal
NLM Journal ID: 101131163
Journal ID: TSWJ

The Scientific World Journal

ISSN: 2356-6140
EISSN: 1537-744X
Publisher: Wiley

ARTICLE INFORMATION
==============================
PMCID: PMC6084158
PMID: 30147482
DOI: 10.1100/tsw.2001.140
Article ID: 750487
Article version: 1
Subjects: Short Report

Molecular Analysis of TTP, a Key Regulator of TNF Activity

Johnson Barbra A. 1 *
Yaffe Michael 2
Blackwell T. Keith 1
1 Department of Pathology, Harvard Medical School and Center for Blood Research, 200 Longwood Ave, Boston, MA 02115, USA
2 Massachusetts Institute of Technology, 77 Massachusetts Avenue, E18-580, Cambridge, MA 02139, USA
*Barbra A. Johnson: johnson@cbr.med.harvard.edu
Publication date: 2001
Electronic publication date: 2001 Feb 16
Volume: 1
First page: 38
Last page: 38
Copyright: Copyright © 2001 Barbra A. Johnson et al.
Copyright year: 2001
License: This is an open access article distributed under the Creative Commons Attribution License, which permits unrestricted use, distribution, and reproduction in any medium, provided the original work is properly cited.
License URL: https://creativecommons.org/licenses/by/3.0/

Funding: U.S. Army 17-94-J-4063 National Institutes of Health PO1 HD17461

==============================
Miami *Nature Biotechnology* Short Reports
*TheScientificWorld* (2001) 1(S3), 38SR
ISSN 1532-2246; DOI 10.1100/TSW.2001.140

### MOLECULAR ANALYSIS OF TTP, A KEY REGULATOR OF TNFα ACTIVITY

Barbra A. Johnson*[,1], Michael Yaffe[2], and T. Keith Blackwell[1]

[1]Department of Pathology, Harvard Medical School and Center for Blood Research, 200 Longwood Ave, Boston, MA 02115; [2] Massachusetts Institute of Technology, 77 Massachusetts Avenue, E18-580, Cambridge, MA 02139

• • * johnson@cbr.med.harvard.edu

**INTRODUCTION.** The immediate-early protein tristetraprolin (TTP) is induced by numerous extracellular stimuli and during some apoptotic events. TTP and the related proteins TIS11b and TIS11d (TTP/TIS11 proteins) are members of a family with highly conserved $Cys_3His$ zinc fingers that are associated with RNA binding. In mice, lack of TTP causes an inflammatory syndrome that is mediated by TNFα 1, and evidence indicates that TTP can bind and destabilize TNFα and other cytokine mRNAs2. *In vitro,* all three TTP/TIS11 proteins possess this activity and it is present within the zinc finger region3. Several precedents suggest however that TTP/TIS11 proteins may also be implicated in survival, growth, or apoptotic pathways; a model we have addressed in this study.

**METHOD.** Blue cell assays were used to measure the extent of apoptosis caused by TTP/TIS11 proteins under different conditions 4. A Yeast 2-Hybrid screen for TTP interactors was carried out using a mouse embryonal cDNA library. Interaction between TTP and 14-3-3 was verified by coimmunoprecipitation in 3T3 and HeLa cells. Binding of TTP to different 14-3-3 isoforms was investigated using lysates expressing TTP, and GST-14-3-3 beads.

**RESULTS.** We have determined that various cell types undergo programmed cell death in response to constitutive low-level TTP/TIS11 protein expression. This cell death involves DNA cleavage, caspase activation, and stimulation of the mitochondrial death machinery, indicating that it proceeds by apoptosis. Mapping experiments revealed that both the zinc finger region and C-terminal domain of TTP are necessary to induce significant apoptosis. Surprisingly, expression of TTP but not TIS11b or TIS11d sensitizes cells to the apoptotic effects of TNFα, 4.

Upon conducting a yeast-2-hybrid analysis to screen for interacting proteins, we identified 14-3-3 as a potential interactor with the C-terminal domain of TTP. We verified that this interaction could also occur in a mammalian system via co-immunoprecipitation of overexpressed TTP and endogenous 14-3-3. All three TTP/TIS11 proteins can bind to GST-14-3-3. Binding is phosphorylation dependent, and different 14-3-3 isoforms appear to bind to a varying pattern of phosphorylated forms of TTP. We have also identified a TTP point mutant with diminished binding to 14-3-3.

**DISCUSSION.** Our findings that modest levels of TTP/TIS11 expression induce apoptosis suggest that all three of these proteins may influence growth or survival pathways. TTP alone however is able to sensitize cells to TNFα mediated cell death, leading us to postulate that different family members may receive or propagate death stimuli in different ways. Additionally, the C-terminal of TTP is required to induce apoptosis along with the zinc fingers that can mediate RNA instability. This shows that more than the minimal unit which mediates RNA effects in transfection experiments is necessary for apoptotic function. These observations raise the possibility that some effects seen in the TTP-/- mouse may arise from the inability of TTP to participate in TNF$\alpha$ downstream apoptotic events. Since the C-terminal region is required for apoptosis, and is somewhat divergent between family members, investigating protein/protein interactions in this C-terminal region may allow us to dissect the control of TTP/TIS11 protein function. Since 14-3-3 is known to be involved in apoptotic regulation, investigating its interaction with members of the TTP/TIS11 family may uncover mechanisms by which these proteins are involved in apoptotic events.

**ACKNOWLEDGEMENTS.** This work was supported by the US Army Breast Cancer program (17-94-J-4063), an NIH grant (PO1 HD17461) and a postdoctoral fellowship award from the Arthritis Foundation.

**REFERENCES.**

1. Taylor, G.A. et al. (1996) Immunity 4, 445-454
2. Lai, W.S. et al. (1999) Mol. Cell. Biol. 19, 4311-4323
3. Lai, W.S., Carballo, E., Thorn, J.M., Kennington, E.A., and Blackshear, P.J. (2000) J. Biol. Chem. 275, 17827-17837
4. Johnson, B.A., Geha, M., and Blackwell, T.K. (2000) Oncogene 19, 1657-1664
